# Association between endolymphatic hydrops severity and audio-vestibular outcomes in Ménière’s disease: A systematic review and meta-analysis of correlation coefficients

**DOI:** 10.1007/s00405-026-10122-1

**Published:** 2026-04-06

**Authors:** Mohamed Mohamed Elmoursy, Maha Abdelgaber Abdellah Aly

**Affiliations:** 1 Audiovestular Medicine Unit, ENT Department, Al-Azhar University, Assuit, Egypt; 2 Ear, Nose and Throat Department, Faculty of Medicine, Associate professor of Audiovestibular Medicine, Assiut University, Assiut, Egypt

**Keywords:** Ménière’s disease, Endolymphatic hydrops, Audio-vestibular outcomes, Correlation

## Abstract

**Background:**

Ménière’s disease (MD) is characterized by episodic vertigo, fluctuating sensorineural hearing loss (SNHL), tinnitus, and aural fullness, with endolymphatic hydrops (EH) considered its key pathological substrate. Despite advances in EH assessment (magnetic resonance imaging [MRI]-based grading and physiologic tests), the strength and consistency of associations between EH severity and audio-vestibular measures remain uncertain. This study aimed to review systematically and meta-analyze correlation coefficients quantifying the relationship between EH severity and audiometric and vestibular measures in adults with MD.

**Methods:**

From inception through September 2025, we searched PubMed, Scopus, Web of Science, and Cochrane for publications associating EH with audio-vestibular outcomes in patients with MD. We independently screened and abstracted relevant data, then assessed bias using National Institutes of Health (NIH) tools. Correlations were Fisher Z-transformed and combined using random-effects models. Heterogeneity (I², τ²) and subgroups (cochlear/vestibular and frequency) were assessed.

**Results:**

Sixteen studies were included. EH severity showed a moderate correlation with poorer hearing with pure-tone audiometry (PTA): cochlear *r* = 0.41 (95% CI 0.34–0.48; I²=0%) and vestibular *r* = 0.42 (0.33–0.51; I²=30.9%). Caloric canal paresis (CP%) correlated weakly (cochlear *r* = 0.14 [0.01–0.26]; vestibular *r* = 0.22 [0.10–0.34]; both I²=0%). Clinical stage correlated weak–moderately (cochlear *r* = 0.33 [0.22–0.43; I²=27.8%]; vestibular *r* = 0.36 [0.22–0.49; I²=38.4%]). Frequency-specific PTA associations were strongest at low/mid bands (cochlear *r* = 0.49/0.45; vestibular *r* = 0.51/0.42) and weaker at high frequencies (cochlear *r* = 0.30; vestibular *r* = 0.34). Disease duration showed weak correlations (cochlear *r* = 0.20 [I²=0%]; vestibular *r* = 0.33 [I²=30.2%]).

**Conclusions:**

The EH severity grades demonstrate moderate correlation with poorer hearing and weak correlation with caloric paresis, and clinical stage shows weak-moderate correlation. Relations are strongest at low and mid frequencies and are more consistent for cochlear than vestibular EH. These observations support EH as an imaging correlate of auditory involvement in MD, particularly for low- and mid-frequency hearing thresholds. However, correlations do not establish causality, and standardized imaging with longitudinal designs is needed to clarify temporal relationships and prognostic utility.

**Supplementary Information:**

The online version contains supplementary material available at https://doi.org/10.1007/s00405-026-10122-1.

## Introduction

Ménière-form disorders do not necessarily arise from the exact etiological origins as “Ménière’s disease” (MD) but do share the in common symptomology of episodic vertigo, tinnitus, and sensorineural hearing fluctuations and thus must be discriminated from idiopathic MD [1] – as current framing does require that the symptoms are “not better accounted for by another vestibular diagnosis [2]. Several vestibular and inner-ear conditions can mimic idiopathic MD and must be excluded using targeted clinical assessment, audiovestibular testing, and imaging [3]. Differentiation relies on history (migraine phenotype, autoimmune clues, syphilis risk), targeted testing (audiometry patterns, caloric/video head-impulse dissociation, vestibular-evoked responses), laboratory/serology, and imaging [4]. Diffusion-weighted magnetic resonance imaging (MRI) can assist in the diagnosis of EH and, to some extent, in distinguishing “mimic” cases from idiopathic cases [4].

MD is a progressive disorder affecting the inner ear, characterized by symptoms including vertigo, aural fullness, tinnitus, acute episodes of hearing loss, and varying degrees of sensorineural hearing loss (SNHL) [5]. Diagnosis is guided by established Bárány Society and AAO-HNS criteria [6, 7]. In the general population, MD has been reported to have an incidence and prevalence of approximately 13 cases per 100,000 and 43–513 cases per 100,000, respectively, though > 45 years of age and female sex carries a large burden [8–10]. The high prevalence of MD and associated SNHL result in an economic burden, MD and attack associated hearing loss can lead to trouble in work productivity, and MD can lead to lost workdays [11]. Increased attack frequency of MD significantly diminishes quality of life [12]. There is a pressing need for MD objective biomarkers to support symptom-based diagnosis and refine disease staging.

EH, or expansion of the endolymphatic space, is the histopathological correlate most commonly connected with MD [13] ever since the earliest reports on the seminal temporal bone by Hallpike et al. [14], and later systematic histology in the beginnings of the twenty-first century [15, 16]. Gadolinium (Gd)-enhanced in vivo MRI can demonstrate EH in cochlear and vestibular compartments with specific delayed post-Gd sequences [17]. Both intratympanic (IT) and intravenous (IV) infusion of Gd have been shown to demonstrate EH with comparable invasiveness and image quality [18, 19]. The most common grading systems, the Nakashima MRI [20] scale for cochlear and vestibular EH, the four-stage vestibular grading (e.g., Bernaerts) [21], and some recent 3D real inversion-recovery and HYDROPS-type protocols [22] have gained popularity in MD diagnosis and staging.

This review is restricted to outcomes related to MRI-graded EH in MD and evaluates associations between EH and pure-tone audiometry (PTA; overall and low-, mid-, and high-frequency bands), canal paresis for caloric testing (CP%), clinical stage, and disease duration. Staging criteria: four-frequency PTA ( induce 0.5–3 kHz) and caloric probe low frequency canal function, classic dissociation with relatively standard video head impulse test (vHIT) emphasizing complementary exam domains as well as the cases for multidimensional evaluation [23]. Reported associations vary across studies, likely reflecting differences in outcome definitions and MRI assessment approaches [24] but tend to mirror clinical audiology classification. Other MRI studies demonstrate positive but variably sized correlations between EH severity and these end points, which may reflect heterogeneity in imaging protocols for MRI (IV vs. IT Gd; 3D- [Fluid-attenuated inversion recovery] FLAIR/3D-real inversion recovery [IR]), grading scales, timing of attacks, laterality, and small sample size. For that reason, it is appropriate to employ a quantitative synthesis combining Fisher z-transformed correlations between the compartments (cochlear vs. vestibular) and frequency bands.

## Methods

### Registration and protocol

A pre-study eligibility criteria and methodological protocol were set and registered with PROSPERO (ID: CRD420251230277). Methodology of this study was adherent to PRISMA statement guidelines [25].

### Information source and search strategy

We searched PubMed, Scopus, Web of Science and Cochrane library for studies fulfilling our criteria published from inception till September 2025. Search strategies combined controlled vocabulary and free-text terms for “endolymphatic hydrops”, “Ménière’s disease” and “inner-ear MRI” that were connected using suitable Boolean operators. References lists were hand-searched for Reference lists were hand-searched, and corresponding authors were contacted for missing data or patient-level results enabling computation of correlation coefficients.

### Eligibility criteria

#### Inclusion criteria

We included human observational studies (cross-sectional, cohort, or case-control) among adults or adolescents or elderly with Meniere’s disease, where EH severity in the cochlea and/or vestibule was measured on inner-ear MRI using any established grading (e.g. Nakashima, Barath/Bernaerts, Gurkov/Yang, He, or continuous % measures). Studies were eligible if at least one prespecified audiovestibular outcome was reported on the same ear or patient as the exposure (including overall PTA, CP [primary outcomes], low (125–500 Hz) / mid (1–2 kHz) / high (4–8 kHz)-frequency PTA, clinical stage, and duration of disease as reported/defined in the original study), and sufficient data were reported to derive a correlation coefficient (Pearson r or Spearman r) between EH and outcome. For bilateral/mixed laterality cohorts, we extracted the correlation as reported and prioritized ear-matched exposure–outcome pairs when available.

#### Exclusion criteria

We excluded non-human studies; non-original reports (reviews, editorials, letters, abstracts without extractable data); very small case series/case reports (< 10); studies lacking MRI-based EH quantification or grading; reports in which exposure and outcome were not linked in the same ear/patient or in which group-level data prevented calculating correlation; studies with insufficient statistics to derive r/v (no author data available); mixed-aetiology cohorts where MD data were not separable; and retracted publications.

### Selection process

Databases search results we exported and uploaded into Rayyan platform [26] for blinded screening of these records. We independently screening studies against eligibility criteria in two steps screening process. First step, they screened titles and abstracts of studies. Second step, screening of retrieved full texts. Any disagreements between the authors were solved by consensus.

### Risk of bias assessment

We evaluated risk of bias using the National Institutes of Health (NIH) instrument for observational cohort and cross-sectional studies [27]. We independently judged selection, exposure (MRI-EH) measurement (reader blinding), validity/timing of outcome (PTA/vestibular), confounding, missing data, and transparency of analysis; differences were resolved by consensus, and overall judgments were Good/Fair/Poor.

### Statistical analysis

We summarized the correlation between EH severity grade and each outcome on the correlation scale: study’s Pearson r (or Spearman p), while available; if r was not available, we (i) recomputed it using patient-level data if possible, or (ii) back-calculated r from reported t/z/F (two-tailed p). A study-by-study summary of how each correlation coefficient was obtained is provided in *Table *S1. We performed separate meta-analysis for cochlear endolymphatic hydrops (cEH) and vEH. We then took the Fisher’s z transformation (yi = atanh(r), vi = 1/(n -3)) [28] and fit random-effects models using package metafor in R (version 4.4.2) [29], reporting pooled r (back transformed) and its 95% confidence interval (CI). Pearson and Spearman coefficients were both treated as correlation effect sizes and synthesized on the Fisher-*z* scale. Interpretations of r wad banded according to Evans (1996) book that stratify r value as following: 0.00–0.19: very weak correlation, 0.20–0.39: weak correlation, 0.40–0.59: moderate correlation, 0.60–0.79: strong correlation and 0.80–1.00: very strong correlation. Because EH severity was reported using different established grading systems (and occasionally quantitative measures such as %EH), we treated these as alternative proxies of the same underlying construct (EH severity). Between-study heterogeneity was quantified using *I*² and τ² tests and interpreted as partly reflecting differences in EH assessment methods or other potential factor. In case of significant heterogeneity, sensitivity analysis was conducted trying to solve this heterogeneity. Subgroup analyses for the primary outcome examined differences by MRI acquisition technique, contrast route (IT vs. IV), and study quality (fair vs. good). For key outcomes, subgrouping by contrast route was prioritized.

## Results

### Study selection

**Fig. 1:**
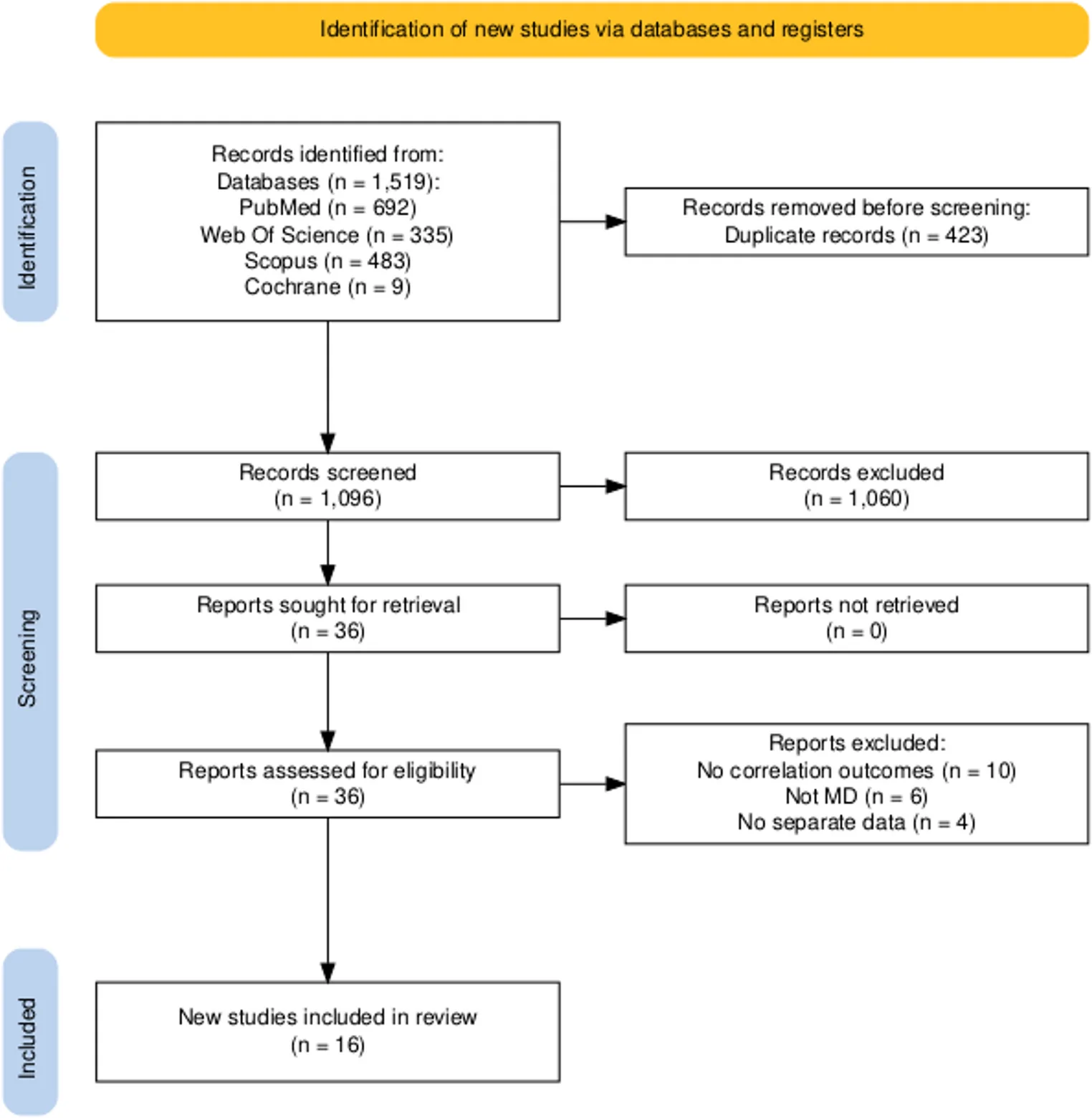
PRISMA flow diagram of selection process

The search on electronic databases yielded a total of 1,519 relevant articles. 423 duplicates were removed. After rigorous screening of the 1,096 remaining articles based on the inclusion and exclusion criteria, 36 articles were included from title and abstract screening. Ultimately, 16 articles [30–45] were eligible for analysis following a meticulous examination of their full text. The detailed flowchart of the screening process is demonstrated in Fig. 1.

### Characteristics of included studies

Sixteen studies (total *n* = 921 patients; median per study 47, range 16–192) were predominantly single-centered, cross-sectional, mostly retrospective in nature, although some were part of prospective/clinical cohorts. Spatially, there were 11 studies in China, two in South Korea, and one each in Poland, Germany, and Turkey. Chronic diagnostic frameworks were mostly 2015 versions of the Bárány Society, although some cohorts used AAO-HNS 1995, and one used AAO-HNS 2020; the majority of cohorts had unilateral MD (including several cohorts which explicitly reported 100% unilateral), one studied bilateral MD (BMD) exclusively, and several were non-specific. The units of analysis were also variable in methods-comparison studies. Age ranges reported started in the early forties and ended in the early sixties (e.g., means/medians = ≈ 43–61 years). Table 1 explicates summary of included studies.

**Table 1:** Summary of the included studies

Study ID	Country	Study design	Population	Sample size	Diagnostic criteria used	MRI acquisition technique (Core sequence / Contrast approach)	Laterality	Age, mean (SD) or median [IQR]	Sex (female, *n* (%)
Choi 2017	Korea	Prospective, cross-sectional	Definite unilateral MD; ≥2 vertigo episodes (> 20 min) in prior 3 months; unilateral aural symptoms; excluded prior intratympanic gentamicin/endolymphatic sac surgery and abnormal vHIT.	16	AAO-HNS 1995	hT2W MR cisternography + hT2W-3D-FLAIR (PPI/PEI) to generate HYDROPS and HYDROPS-Mi2 / IV gadolinium–enhanced inner-ear MRI	Unilateral MD (100%)	NR	4 (25%)
Han 2023	China	Retrospective, cross-sectional	MD per Bárány 2015; completed PTA before endolymphatic sac decompression; inner-ear gadolinium MRI (3D-real IR) within 1 month; excluded 3D-FLAIR scans, repeat exams, late MRI, prior head/neck surgery, poor image quality.	31 patients (59 ears)	Bárány Society 2015	3D-real IR / intratympanic diluted Gd-DTPA	NR	60 [51–65]	19 (61.3%)
Han 2024	China	Retrospective, cross-sectional	MD per Bárány 2015; PTA and gadolinium inner-ear MRI within 1 month (pre-surgery); excluded 3D-FLAIR scans, poor quality imaging, MRI > 1 month after symptom onset; ethics approved.	30 patients; 58 ears	Bárány Society 2015	3D-real IR / intratympanic Gd-DTPA	NR	43 (9.1)	20 (66.7%)
Hu 2023	China	Retrospective, cross-sectional	Unilateral definite or probable MD (2015 Bárány); excluded major systemic illness, spine trauma, middle-ear disease, VS, barotrauma, LVAS or other congenital malformations.	70	Bárány Society 2015	3D-real IR/ bilateral intratympanic gadolinium administration	Unilateral (100%)	50.8 (11.8)	33 (47.1%)
Huang 2023	China	Cross-sectional	MD per 2015 guidelines; no prior treatment before enrollment; 3D-real IR MRI and PTA	54 patients (103 ears)	Bárány Society 2015	3D-real IR/ intratympanic gadopentetate	Unilateral 44/54 (81.5%); bilateral 10/54 (18.5%).	46.76 (10.23)	33 (61.1%)
Jasinska 2022	Poland	Cross-sectional	Patients with unilateral definite MD; MRI and audio-vestibular tests off-attack.	38	AAO-HNS and Bárány Society guidelines	3D-FLAIR (pre + 4-hour delayed) / IV gadobutrol	Unilateral 100%	54.39	19 (50.0%)
Jerin 2018	Germany	Prospective observational	Consecutive clinically definite MD; intratympanic LEIM; audiometry and caloric 1 day prior; attack diaries.	192	AAO-HNS 1995	LEIM (locally enhanced inner ear MRI) / intratympanic contrast application	NR	54.3 [15–79]	88 (45.8%)
Kirbac 2022	Turkey	Cross-sectional	Adults with unilateral definite MD; IV Gd inner-ear MRI, PTA, vHIT, cVEMP, caloric, DHI.	16	Definite unilateral MD; formal criteria not explicitly named	3D-FLAIR / IV gadobutrol	Unilateral 100%	41 (9.7)	7 (43.8%)
Leng 2023	China	Retrospective cohort	Patients with unilateral definite MD after intratympanic Gd MRI.	70	AAO-HNS 1995	3D-FLAIR / intratympanic MultiHance	Unilateral 100%	50.7	42 (60.0%)
Liu 2021	China	Retrospective	patients with unilateral definite MD per Bárány (2015).	69	Bárány Society 2015	3D-FLAIR + 3D-T2-SPAIR / intratympanic Omniscan	Unilateral 100%	52.62 (12.11)	35 (50.7%)
Liu 2024	China	Retrospective cross-sectional	BMD diagnosed 2016–2023; each ear assessed; excluded poor MRI quality or PTA non-response.	77 patients (154 ears)	Bárány Society 2015	T2-SPACE plus an inversion-recovery hydrops sequence / IV gadolinium	Bilateral 100%	50.9	33 (42.9%)
Oh 2021	South Korea	Cross-sectional observational	Adults meeting Bárány MD criteria; concurrent VM and VM-MD cohorts also enrolled (comparative study).	29 patients	Bárány Society criteria 2015	MR cisternography (MRC) + hT2W 3D-FLAIR / IV gadolinium	NR	61.1 (9.5)	19 (65.5%)
Wang 2025	China	Retrospective cohort	Unilateral MD (DMD or PMD) per AAO-HNS 2020; excluded bilateral MD, insufficient Gd uptake, IV Gd, otitis media, head trauma.	111 patients (DMD = 91)	AAO-HNS 2020	3D-FLAIR / Intratympanic gadolinium	Unilateral 100%	56 [45–63]	DMD: 45 (49.45%)
Wu 2016	China	Prospective cross-sectional	Unilateral definite MD; ≥2 vertigo episodes with HL, tinnitus, aural fullness; excluded middle-ear or neurologic disorders.	54 unilateral definite MD patients	AAO-HNS 1995	T2-SPACE + 3D-FLAIR + inversion-recovery hydrops sequence / Intratympanic gadolinium	Unilateral 100%	52 [23–74]	9 (16.7%)
Zhang 2021	China	Prospective clinical study	Unilateral MD; excluded Gd allergy, neurologic deafness, otitis media, ear surgery.	24 patients	Bárány Society 2015	3D-FLAIR / IV double-dose gadolinium	Unilateral 100%	54 [21–74]	13 (54.2%)
Zhang 2022	China	Retrospective cross-sectional	Definite unilateral MD; no Gd allergy, neurologic deafness, otitis media, or ear surgery.	40 patients	AAO-HNS 2015	3D-FLAIR / IV double-dose gadolinium	Unilateral 100%	60 [33–77]	17 (42.5%)

*AAO-HNS* American Academy of Otolaryngology–Head and Neck Surgery, *AFP* Alpha-fetoprotein, *BMD* Bilateral Ménière’s disease, *cVEMP* Cervical vestibular evoked myogenic potentials, *DHI* Dizziness Handicap Inventory, *DMD* Definite Ménière’s disease, *FLAIR* Fluid-attenuated inversion recovery, *Gd* Gadolinium, *HL* Hearing loss, *IR* Inversion recovery, *IV* Intravenous, *IQR* Interquartile range, *LEIM* Low-energy intratympanic MRI, *LVAS* Large vestibular aqueduct syndrome, *MD* Ménière’s disease, *MRI* Magnetic resonance imaging, *NR* Not reported, *PMD* Probable Ménière’s disease, *PTA* Pure-tone audiometry, *vHIT* Video head impulse test, *VM* Vestibular migraine, *VM-MD* Vestibular migraine with coexisting Ménière’s disease, *VS* Vestibular schwannoma, *Gd-DTPA* Gadolinium-Diethylenetriaminepentaacetic acid

### Risk of bias assessment

Overall quality was Good in eight studies and Fair in eight others. Outstanding qualities included well-defined objectives, populations, and the uniform evaluation of EH and related results. Recurrent weaknesses included absent justifications for sample sizes, lack of blinding for outcome assessors, evaluations of exposure performed only at one point in time (or for short periods), and confounder adjustments which in some cases could lead to underestimating or overestimating the strength of the correlation. Table 2 shows detailed summary of risk of bias assessment of included studies.

**Table 2:** Risk of bias assessment of the included studies using NIH quality assessment scale

Study	Q1	Q2	Q3	Q4	Q5	Q6	Q7	Q8	Q9	Q10	Q11	Q12	Q13	Q14	Overall Quality
Huang 2023	√	X	X	√	X	√	√	√	√	√	√	√	√	√	Fair
Jasińska 2022	√	X	√	√	√	√	√	√	√	√	√	√	√	√	Good
Jerin 2018	√	√	√	√	√	√	√	√	√	√	√	X	√	√	Good
Kirbac 2022	√	X	√	√	X	√	√	√	√	√	√	√	√	√	Fair
Leng 2023	√	√	√	√	√	√	X	X	X	√	X	X	X	√	Fair
Liu 2021	√	√	√	√	√	√	√	X	√	√	√	X	√	√	Fair
Choi 2017	√	√	√	√	√	√	X	√	√	√	√	√	√	√	Good
Han 2023	√	√	√	√	√	√	√	√	√	√	√	√	X	√	Good
Han 2024	√	√	√	√	√	√	√	X	√	X	X	√	√	√	Fair
Hu 2023	√	√	√	√	√	√	X	√	√	√	√	√	√	√	Good
Liu 2024	√	√	√	√	√	√	√	√	√	√	√	√	√	√	Good
Oh 2021	√	X	√	√	√	√	√	X	√	√	√	X	X	√	Fair
Wang 2025	X	√	X	√	√	√	√	√	√	√	X	√	√	√	Fair
Wu 2016	√	X	√	√	X	√	X	√	√	√	X	√	√	√	Fair
Zhang 2021	X	√	√	√	√	√	√	√	√	√	√	√	√	√	Good
Zhang 2022	√	√	√	√	√	√	X	√	√	√	√	X	√	√	Good

Q1. Was the research question or objective in this paper clearly stated?/ Q2. Was the study population clearly specified and defined?/ Q3. Was the participation rate of eligible persons at least 50%?/ Q4. Were all the subjects selected or recruited from the same or similar populations (including the same time period)? Were inclusion and exclusion criteria for being in the study prespecified and applied uniformly to all participants?/Q5. Was a sample size justification, power description, or variance and effect estimates provided?/ Q6. For the analyses in this paper, were the exposure(s) of interest measured prior to the outcome(s) being measured?/ Q7. Was the timeframe sufficient so that one could reasonably expect to see an association between exposure and outcome if it existed?/ Q8. For exposures that can vary in amount or level, did the study examine different levels of the exposure as related to the outcome (e.g., categories of exposure, or exposure measured as continuous variable)?/ Q9. Were the exposure measures (independent variables) clearly defined, valid, reliable, and implemented consistently across all study participants?/ Q10. Was the exposure(s) assessed more than once over time?/ Q11. Were the outcome measures (dependent variables) clearly defined, valid, reliable, and implemented consistently across all study participants?/ Q12. Were the outcome assessors blinded to the exposure status of participants?/ Q13. Was loss to follow-up after baseline 20% or less?/ Q14. Were key potential confounding variables measured and adjusted statistically for their impact on the relationship between exposure(s) and outcome(s)?

### Primary outcomes

#### Overall pure tone audiometry (PTA)

**Fig. 2:**
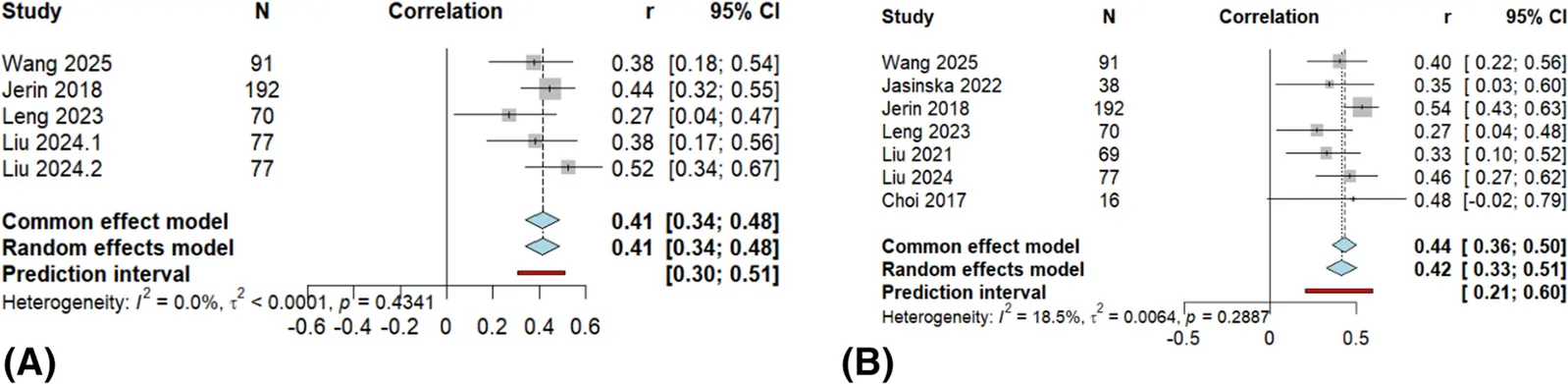
Forest plot of meta-analysis of correlation between EH and PTA; (**A**) cochlear EH, (**B**) Vestibular EH

Using a random effect model of Fisher-z-transformed correlations between EH severity grade and PTA, EH severity was significantly and positively associated with poorer hearing in both compartments. For cochlear EH pooling five effect sizes yielded a moderate correlation with *r* = 0.41 (95% CI [0.34, 0.48]) with no detected heterogeneity (τ^2^ < 0.0001, I^2^ = 0%) (Fig. 2a). Fore vestibular EH, pooled analysis showed also a moderate correlation between EH severity grade and PTA (*r* = 0.42, 95% CI [0.33, 0.51]) with low to moderate heterogeneity (τ² = 0.0064, I² = 18.5%) (Fig. 2b).

#### Subgroup analysis

For cochlear EH (cEH), subgrouping by injection route showed moderate correlations for both intratympanic (IT) (*r* = 0.39) and intravenous (IV) (*r* = 0.46) administration (*Fig. *S1*a*). When analyzed by MRI sequence, correlations ranged from *r* = 0.33 for 3D-FLAIR to *r* = 0.46 for SPACE + IR hydrops (*Fig. *S2*a*). Further categorization by clinical diagnostic criteria yielded correlations between *r* = 0.38 for AAO-HNS 1995 and *r* = 0.46 for Bárány Society 2015 (*Fig. *S3*a*). Across all cEH subgroups, there were no statistically significant differences, with p-values for subgroup differences ranging from 0.349 to 0.730. Heterogeneity remained consistently low, with I^2^ values between 0% and 12.3%. Stratification by study quality demonstrated consistent correlations between fair-quality (*r* = 0.33) and good-quality (*r* = 0.45) studies with *p* = 0.054 (*Fig. *S4*a*).

For vestibular EH (vEH), the correlation with hearing loss remained stable across injection routes, with an IT correlation of *r* = 0.44 and an IV correlation of *r* = 0.43 (*Fig. *S1*b*). Stratification by MRI sequence showed a wider range of correlations, from *r* = 0.34 for 3D-FLAIR to *r* = 0.54 for LEIM (*Fig. *S2*b*). Subgrouping by diagnostic criteria showed results between *r* = 0.39 and *r* = 0.47 (*Fig. *S3*b*). While subgroup differences were not significant (*p* > 0.05), heterogeneity was more variable in the vEH analysis. Specifically, the IT subgroup and the AAO-HNS criteria subgroup showed higher heterogeneity at I^2^ = 56.1% and 60.2%, respectively, whereas the IV and Bárány Society subgroups showed no heterogeneity (I^2^ = 0%). In terms of study quality, good-quality studies showed a stronger correlation (*r* = 0.50) compared to fair-quality studies (*r* = 0.34) with a statistically significant difference observed based on study quality (*p* = 0.0026) (*Fig. *S4*b*).

#### Caloric canal paresis (CP %)

**Fig. 3:**
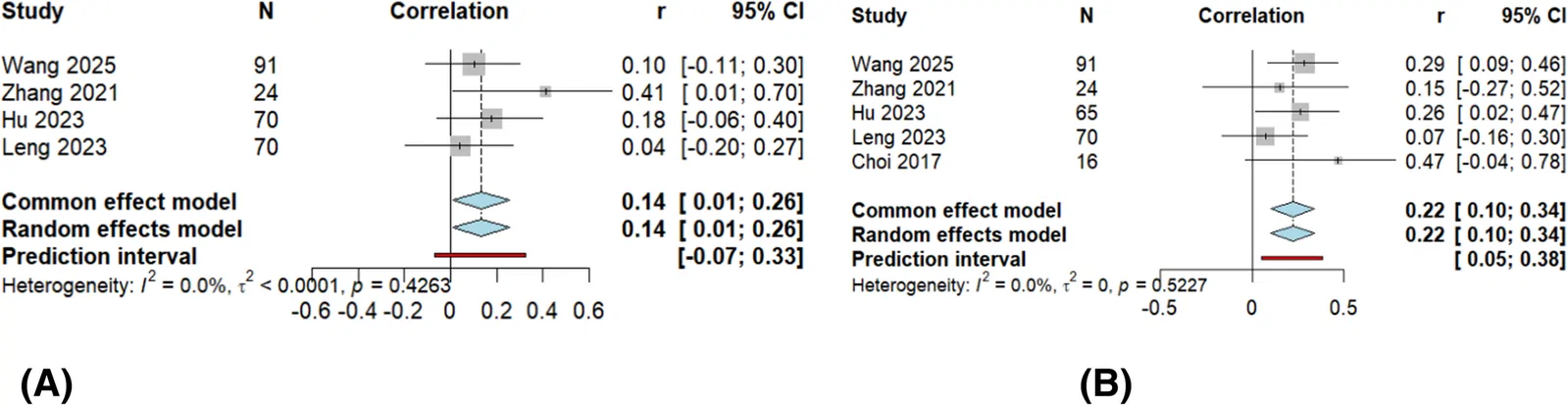
Forest plot of meta-analysis of correlation between EH and CP; (**A**) cochlear EH, (**B**) Vestibular EH

CP showed small correlation with EH according to pooled analysis. For cochlear EH, meta-analysis showed very week association between cEH and CP (*r* = 0.14, 95% CI [0.01, 0.26]) with no heterogeneity (τ^2^ = 0.00, I^2^ = 0%) with no difference between IV and IT groups (*p* = 0.13) (*Fig. *S5*a*). Vestibular EH showed higher but still week correlation with CP (*r* = 0.22, 95% CI [0.10, 0.34]) with no heterogeneity (τ^2^ = 0.00, I^2^ = 0%) with no differences between IV and IT (*Fig. *S5*b*). Forest plots are illustrated in Fig. 3.

### Secondary outcomes

#### MD clinical stage

**Fig. 4:**
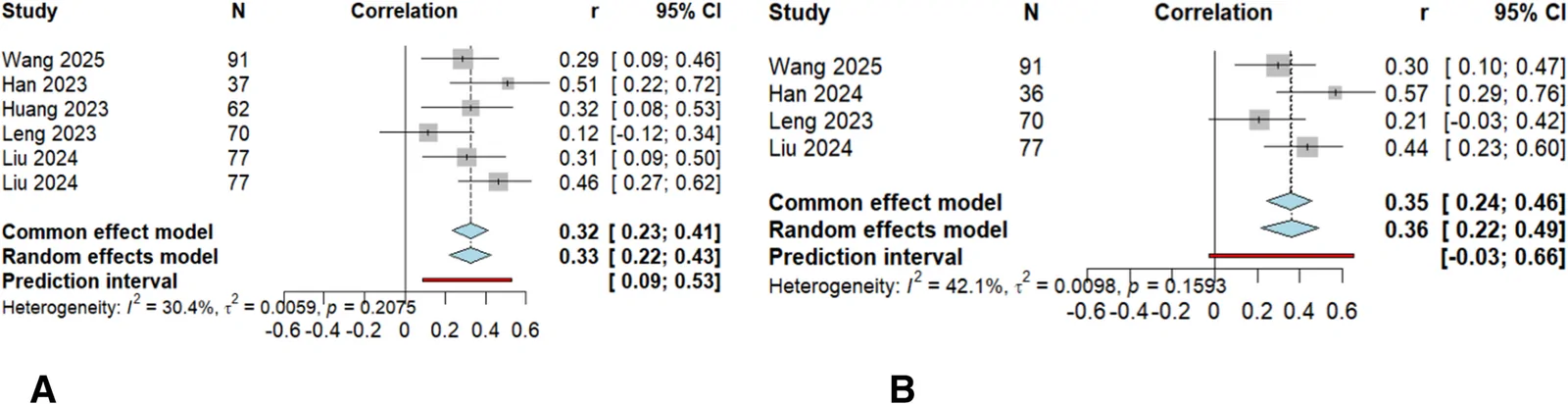
Forest plot of meta-analysis of correlation between EH and disease clinical stage; (**A**) cochlear EH, (**B**) Vestibular EH

Meta-analysis showed positive weak to moderate correlation between MD clinical stage and cEH and vEH (*r* = 0.33, 95% CI [0.22, 0.43]) and (*r* = 0.36, 95% CI [0.22, 0.49]) respectively. Heterogeneity analysis showed non-significant heterogeneity in cEH (τ^2^ = 0.00591, I^2^ = 30.4%) and in vEH (τ^2^ = 0.00985, I^2^ = 42.1%). (Fig. 4)

For cochlear EH (cEH), subgroup analysis by injection route showed a moderate positive correlation between the degree of hydrops and the clinical stage of Meniere’s disease. Both intratympanic (IT) and intravenous (IV) administration routes yielded similar results, with a correlation of *r* = 0.29 for IT and *r* = 0.39 for IV. There was no statistically significant difference between these two subgroups (*p* = 0.3802). Heterogeneity remained relatively low to moderate, with an I^2^ of 35.9% in the IT group and 20.1% in the IV group (*Fig. *S6*a*).

For vestibular EH (vEH), the subgroup analysis by injection route also demonstrated a stable correlation with clinical stage regardless of the administration method. The correlation for the IT subgroup was *r* = 0.34, while the IV subgroup showed a correlation of *r* = 0.36. No significant subgroup difference was detected between the two routes (*p* = 0.5084). In terms of heterogeneity, the IT subgroup exhibited moderate levels (I^2^ = 52.6%), whereas no heterogeneity was reported for the IV subgroup as it was based on a single study (*Fig. *S6*b*).

#### Frequency-specific PTAs

**Fig. 5:**
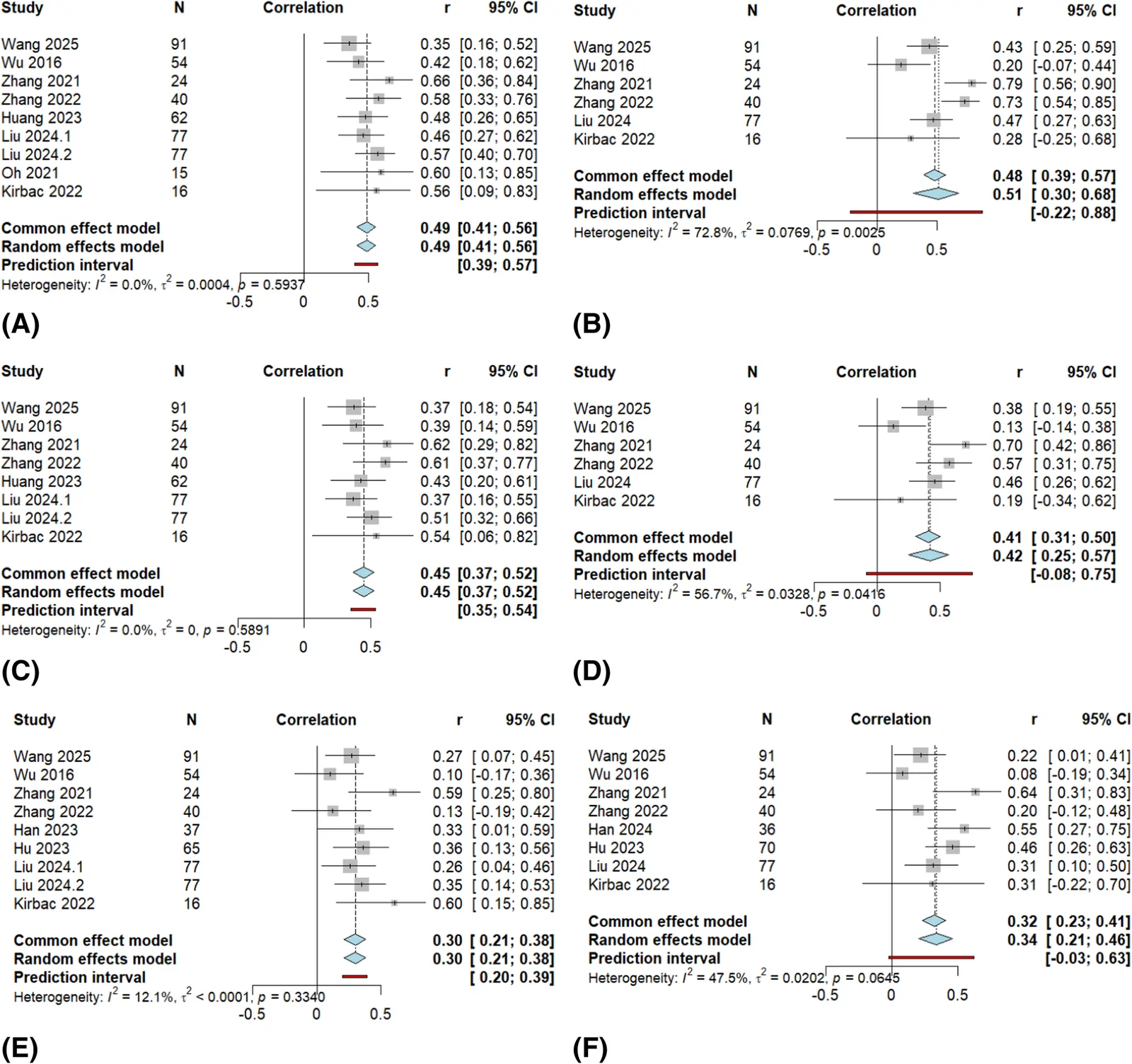
Forest plots of meta-analysis of correlation between EH and specific frequencies PTA; (**A**) low frequency PTA; cochlear EH, (**B)** low frequency PTA; vestibular EH, (**C**) Mid frequency PTA; cochlear EH, (**D**) Mid frequency PTA; vestibular EH, (**E**) High frequency PTA; cochlear EH, (**F**) High frequency PTA; vestibular EH

In the frequency-specific analyses, cochlear EH was moderately and consistently associated with hearing loss at low and mid bands and less strongly associated at high frequencies: low-frequency (k = 9) pooled *r* = 0.49 (95% CI [0.41, 0.56], I^2^ = 0%), mid-frequency (k = 8) *r* = 0.45 (95% CI [0.37, 0.52], I^2^ = 0.0%), high-frequency (k = 9) *r* = 0.30 (95% CI [0.21, 0.38], I^2^= 12). vEH was similar in magnitude but more heterogeneous: low-frequency (k = 6) *r* = 0.51 (95% CI [0.30, 0.68], I^2^ = 72.8%), mid-frequency (k = 6) *r* = 0.42 (95% CI [0.25, 0.57], I^2^ = 56.7%) and high-frequency (k = 8) *r* = 0.34 (95% CI [0.21, 0.46], I^2^= 47.5%). Overall, the associations are strongest at low/mid frequencies and are more consistent for cochlear than vEH. (Fig. 5)

#### Duration of the disease

**Fig. 6:**
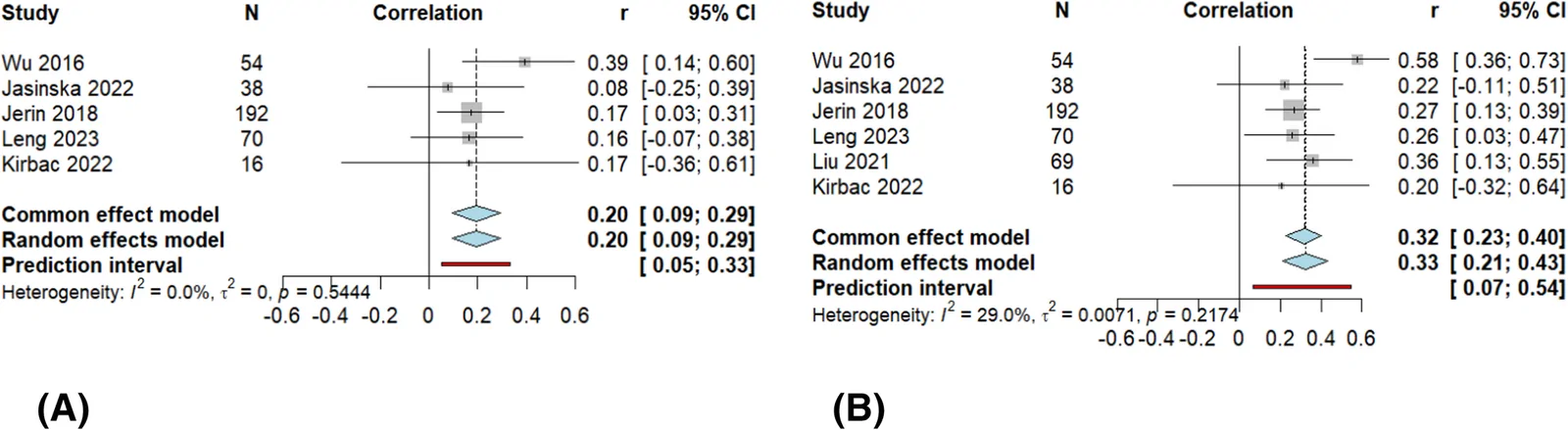
Forest plot of meta-analysis of correlation between EH and disease duration; (**A**) cochlear EH, (**B**) Vestibular EH

The association between disease duration and EH severity was weak in both compartments: cochlear *r* = 0.20 (95% CI 0.09–0.29) with no heterogeneity (I² = 0.0%), and vestibular *r* = 0.33 (95% CI 0.21–0.43) with low–moderate heterogeneity (I² = 29%). Although higher for vestibular than cochlear, the correlation remains weak. (Fig. 6)

## Discussion

### Main findings and previous research

Meta-analysis revealed a moderate and consistent correlation between MRI-graded EH severity and overall PTA in both the cochlear and vestibular compartments. In practice, the higher the hydropic burden, the worse the hearing thresholds were, and the relationship remained even when hydrops was measured in the vestibular labyrinth rather than only in the cochlea. This pattern suggests that EH is not merely an incidental imaging finding but is associated with clinically relevant auditory dysfunction, associated with clinically relevant auditory dysfunction, and is compatible with clinicopathological models linking labyrinthine fluid imbalance to the characteristic fluctuating SNHL of MD [46]. The consistency of this correlation across various routine MRI acquisition methods and contrast administration routes supports the applicability of the EH–hearing connection to routine clinical practice. For the majority of subgroup strata, the consistency of effect direction and the absence of other cross-study variability further substantiate the conclusion made. Notably, the association was more stable for cochlear imaging than for vestibular imaging, indicating a tighter coupling between within-cochlear structural changes and the behavioral fixation of threshold.

Prior diagnostic meta-analyses of imaging studies have shown that cochlear descriptors such as perilymphatic enhancement and MRI-graded EH best discriminate MD from non-MD, providing further support for our interpretation that EH is an indicator of clinically significant auditory pathology [47]. The consistency of relationships between EH and PTA across imaging methods is consistent with the literature, strengthening the claim that EH is a clinically useful and adjunct imaging biomarker rather than merely a descriptive feature of radiology.

Meta-analysis revealed a weak relation between EH severity and the CP% on the caloric test. Such a dissociation is physiologically consistent. Caloric testing probes only a small, low-frequency range of horizontal semicircular canal (HSC) function, requires thermally induced endolymph convection, and is filtered by central velocity storage mechanisms. In contrast, delayed post-contrast MRI gives a relatively static picture of the endolymph volume at a single time point [48]. Vestibular function in the affected ear of MD varies greatly with attack, compensation, and adaptation; thus, a structural metric captured interictally will only loosely represent a dynamic physiologic response. This dissociation persisted across contrast-administration routes, underscoring that EH grading should not be interpreted as a surrogate for caloric responses. The clinical corollary is that, although useful for describing the inner-ear fluid state, EH grading should never be considered a surrogate for caloric results. Comprehensive vestibular phenotyping remains necessary when the focus is on canal-function-based decisions. A systematic review comparing hydrops MRI with audiovestibular testing found only a moderate correlation with cVEMP and a variable correlation with calorics, consistent with our conclusion that EH is not a surrogate for CP [49].

Meta-analysis revealed a weak-to-moderate correlation between EH severity and clinical stage. This is to be expected as current staging schemes are based principally on hearing thresholds; EH affects staging indirectly through its interaction with PTA [36, 50]. The incomplete overlap is, however, also suggestive: clinical stage reflects broader aspects of disease burden (frequency and severity of vertigo attacks, fluctuations, and functional handicap) that a structural imaging measure cannot fully capture. Thus, EH appears to best serve as an additive biomarker to enhance, rather than substitute for, symptom-based staging. In practice, imaging can improve risk communication and follow-up planning but should be considered within a multiparametric, patient-level context.

When examined by route of administration, the stage association remained broadly similar, supporting the conclusion that EH captures a structural component of disease burden that complements symptom-anchored staging. Non-contrast and contrast MRI series have higher EH visualization rates at more advanced stages and comparable diagnostic performance across MD classifications, consistent with our finding of a partial stage relationship, but should be interpreted with the understanding that staging encompasses constructs beyond imaging [51]. This will allow EH to be considered an adjunct, not a substitute, to symptom-anchored staging.

Meta-analysis revealed that the EH-PTA association was most pronounced for low and mid frequency ranges with modest associations at high frequency and with greater internal consistency for cochlear than vestibular EH. This frequency gradient reflects the natural history of Meniere’s disease: apical cochlear locations, which encode low-frequency information, are preferentially affected by hydropic expansion and altered endolymphatic dynamics early in the disease [52, 53], but more basal locations only later. Apical segments are mechanically less stiff and may be more sensitive to volume-pressure perturbations; the consequent distortion of endocochlear potential and hair-cell transduction enhances threshold elevation at low and mid frequencies [54]. A closer match between cochlear imaging grades and frequency-specific PTA confirms the benefit of a compartment-aware interpretation of MRI. It suggests that low- and mid-frequency thresholds are the most sensitive functional readouts of hydropic stress in clinical practice.

Meta-analysis revealed only a modest correlation between disease duration and EH severity across the two compartments. Duration of treatment is a crude measure of cumulative hydropic load in Meniere’s disease; however, because the disorder is variable, evolves heterogeneously, and is altered by therapy and the patient’s behavior, duration is not a reliable indicator of cumulative hydropic load [55]. While some affected persons develop significant hydrops early on, others have recurrent radiologic normalization with persistent clinical symptoms [56]. Cross-sectional study designs further impede temporal inference; MRI is often acquired without standardized timing relative to vertigo spells, and many datasets do not distinguish active from quiescent phases. Therefore, time from onset should not be used to impute imaging severity or to guide prognosis in place of direct measurement. Clinicians should prioritize up-to-date ear-level measurements (imaging plus audiovestibular testing) over calendar time.

Longitudinal imaging suggests that EH can vary over relatively short periods and following interventions, which is one reason why simple elapsed time correlates poorly with structural burden. In addition, analysis of temporal changes indicates inconsistent directional trends in EH evolution, supporting our caution against using duration as an index of severity [57].

Taken together, the overall message is simple. Meta-analysis demonstrated that the EH severity correlates significantly with hearing status (most clearly at low and mid frequencies), correlates poorly with caloric hypofunction, and correlates partially with clinical stage. EH thus serves as a plausible imaging biomarker of the auditory component of MD and as an ancillary, but not conclusive, biomarker of disease severity. It must be stressed that these associations are probabilistic: EH is neither necessary nor sufficient for any given audiovestibular deficit in any ear, and management decisions must combine imaging with careful functional testing and clinical course.

### Limitations

Methodologically, the evidence base is characterized by single-center observational studies with variable sampling frames, inconsistent adjustment for confounders (e.g., age, laterality, active vs. quiescent phase, exposure to treatment), and heterogeneous MRI protocols and grading scales. Moreover, EH severity was synthesized across studies that employed different grading systems and MRI evaluation protocols, and, in some reports, quantitative indices (e.g., %EH). Although these measures reflect the same underlying construct, differences in grading thresholds and granularity may have introduced measurement-related heterogeneity and could have attenuated or inflated the pooled correlations. Notably, for our primary and most clinically important outcomes, between-study heterogeneity was not statistically significant, supporting the robustness of the pooled estimates despite variation in EH assessment methods. In addition, the timing of imaging relative to vertigo attacks was rarely standardized, blinding of outcome assessors was uncommon, and bilateral involvement and ear-level dependence were not routinely addressed. Besides, Laterality-based subgrouping (unilateral vs. bilateral MD) was not feasible due to highly imbalanced and incompletely reported subtype data across studies. In addition, across studies, vestibular test paradigms and audiometric frequency bins were not comparable, making harmonization challenging.

### Clinical implications

MRI-graded EH may provide adjunctive information when counseling on prognostic outcomes for hearing loss, specifically in the low and mid frequencies, and may assist in refining staging and trial stratification. However, in primary care, EH grading should not replace vestibular assessments when clinically indicated, and clinicians are cautioned against relying on imaging to determine vestibular dysfunction.

## Conclusions

MRI-graded EH shows a moderate, clinically relevant association with hearing thresholds in MD, greatest at low and mid frequencies, and only a weak correlation with caloric CP; clinical stage correlates to an intermediate extent. These data suggest that EH may be useful as an ancillary imaging biomarker of auditory involvement, but that EH should not be used without caution to infer vestibular impairment. Standardized imaging protocols, harmonized vestibular test batteries, and longitudinal designs that link imaging to the timing of symptoms are necessary to elucidate causal pathways and fine-tune the prognostic use of EH in the clinic.

## Supplementary Information

Below is the link to the electronic supplementary material.

Supplementary file1 (DOCX 2.90 MB)

## Data Availability

The datasets used and/or analyzed during the current study are in the main manuscript.

## Abbreviations

AAO: HNS–American Academy of Otolaryngology–Head and Neck Surgery
BMD: Bilateral Ménière’s disease
CI: Confidence interval
CP: Canal paresis
cEH: Cochlear endolymphatic hydrops
cVEMP: Cervical vestibular evoked myogenic potential
DHI: Dizziness Handicap Inventory
EH: Endolymphatic hydrops
FLAIR: Fluid–attenuated inversion recovery
Gd: Gadolinium
HSC: Horizontal semicircular canal
HYDROPS: Hybrid of reversed image of positive endolymph signal
IR: Inversion recovery
I²: I–squared heterogeneity statistic
IV: Intravenous
IT: Intratympanic
IQR: Interquartile range
k: Number of effect sizes
MD: Ménière’s disease
MRI: Magnetic resonance imaging
NIH: National Institutes of Health
PTA: Pure–tone audiometry
PRISMA: Preferred Reporting Items for Systematic Reviews and Meta–Analyses
r: Correlation coefficient
SE: Standard error
SNHL: Sensorineural hearing loss
vEH: Vestibular endolymphatic hydrops
vHIT: Video head impulse test
VM: Vestibular migraine
VM: MD–Vestibular migraine with coexisting Ménière’s disease
VS: Vestibular schwannoma
