# Supplementary material for: Association between endolymphatic hydrops severity and audio-vestibular outcomes in Ménière’s disease: A systematic review and meta-analysis of correlation coefficients
Source: Eur Arch Otorhinolaryngol. 2026 Apr 6;283(7):4117–30. doi: 10.1007/s00405-026-10122-1 (PMC13388643; doi:10.1007/s00405-026-10122-1)
Supplement: Supplementary file 1 — Supplementary file1 (DOCX 2.90 MB) [file 405_2026_10122_MOESM1_ESM.docx]

Table S1. Summary of correlation reporting and data derivation methods across included studies

| Study | Method of correlation reporting | Methods of transforming |
| --- | --- | --- |
| **Huang 2023** | Spearman correlation / directly reported |  |
| **Jasińska 2022** | Spearman correlation / p value | The correlation coefficient was approximated by inverting the correlation significance test (p-to-r conversion via t-distribution) |
| **Jerin 2018** | Spearman correlation / directly reported |  |
| **Kirbac 2022** | Spearman correlation | Rebuilt from induvial data reported in Table 1a |
| **Leng 2023** | Spearman correlation / directly reported |  |
| **Liu 2021** | Spearman correlation / directly reported |  |
| **Choi 2017** | Spearman correlation / directly reported |  |
| **Han 2023** | Spearman correlation / directly reported |  |
| **Han 2024** | Spearman correlation / directly reported |  |
| **Hu 2023** | Spearman correlation / directly reported |  |
| **Liu 2024** | Spearman correlation / directly reported |  |
| **Oh 2021** | Spearman correlation / directly reported |  |
| **Wang 2025** | Spearman correlation / directly reported |  |
| **Wu 2016** | Spearman, Pearson correlation tests^*^ / directly reported |  |
| **Zhang 2021** | Spearman correlation / directly reported |  |
| **Zhang 2022** | Spearman correlation | Rebuilt from induvial data reported in Table 1 |

^*^ : the study didn’t mention directly which outcomes were assessed using Pearson and which with Spearman and reported only “Pearson/Spearman r”


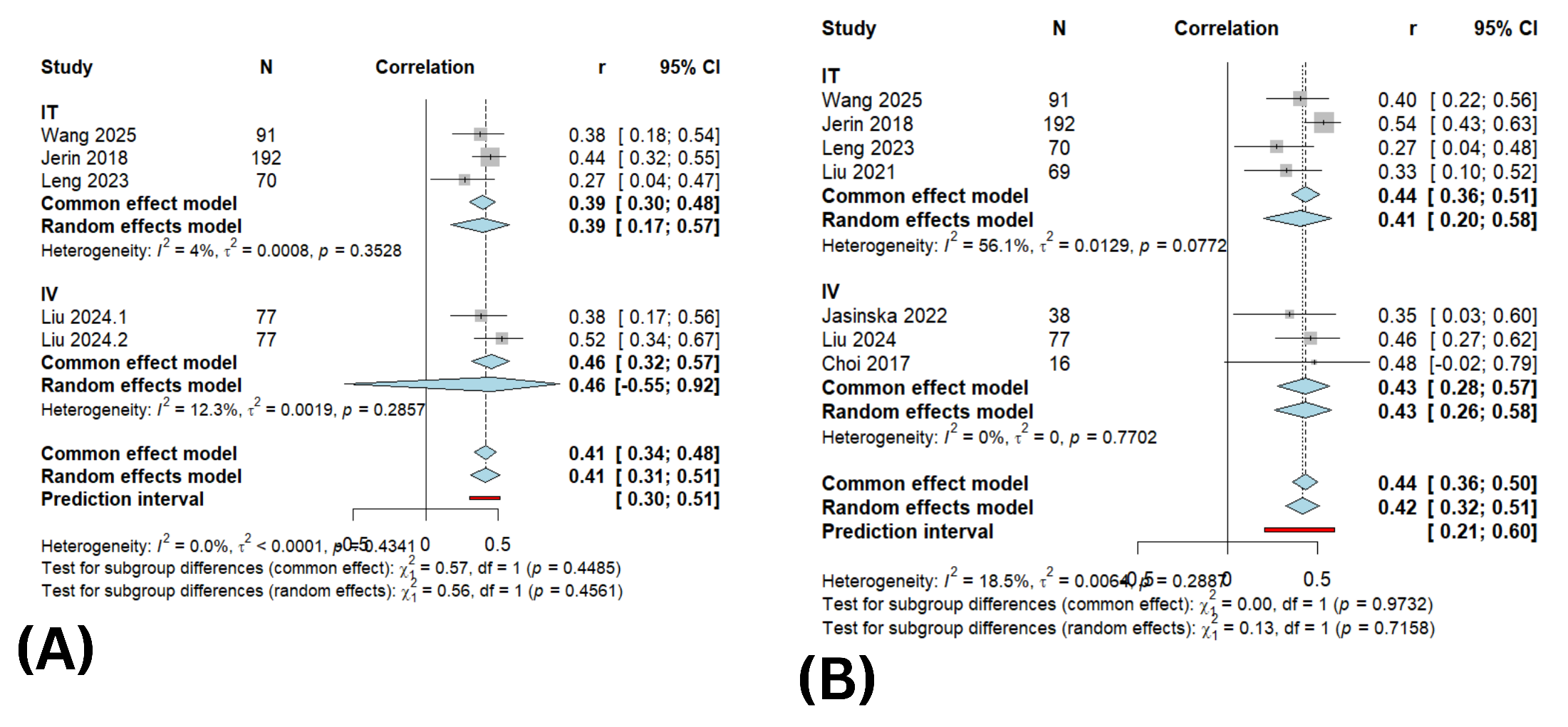


**Figure S1.** Subgroup forest plots of the correlation between EH severity and overall PTA stratified by contrast administration route (IT vs IV): (a) cochlear EH; (b) vestibular EH.


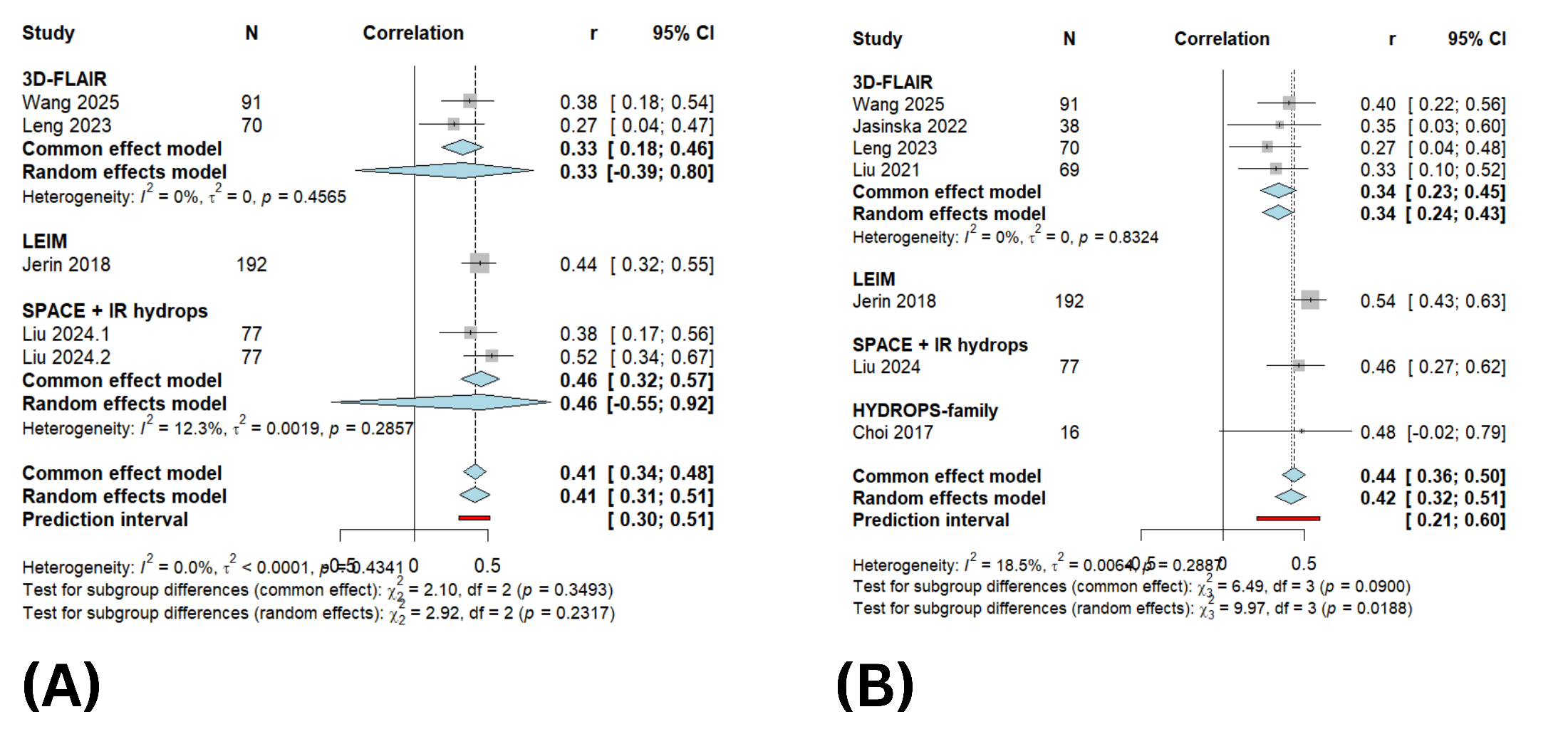


**Figure S2.** Subgroup forest plots of the correlation between EH severity and overall PTA stratified by MRI acquisition sequence/technique: (a) cochlear EH; (b) vestibular EH.


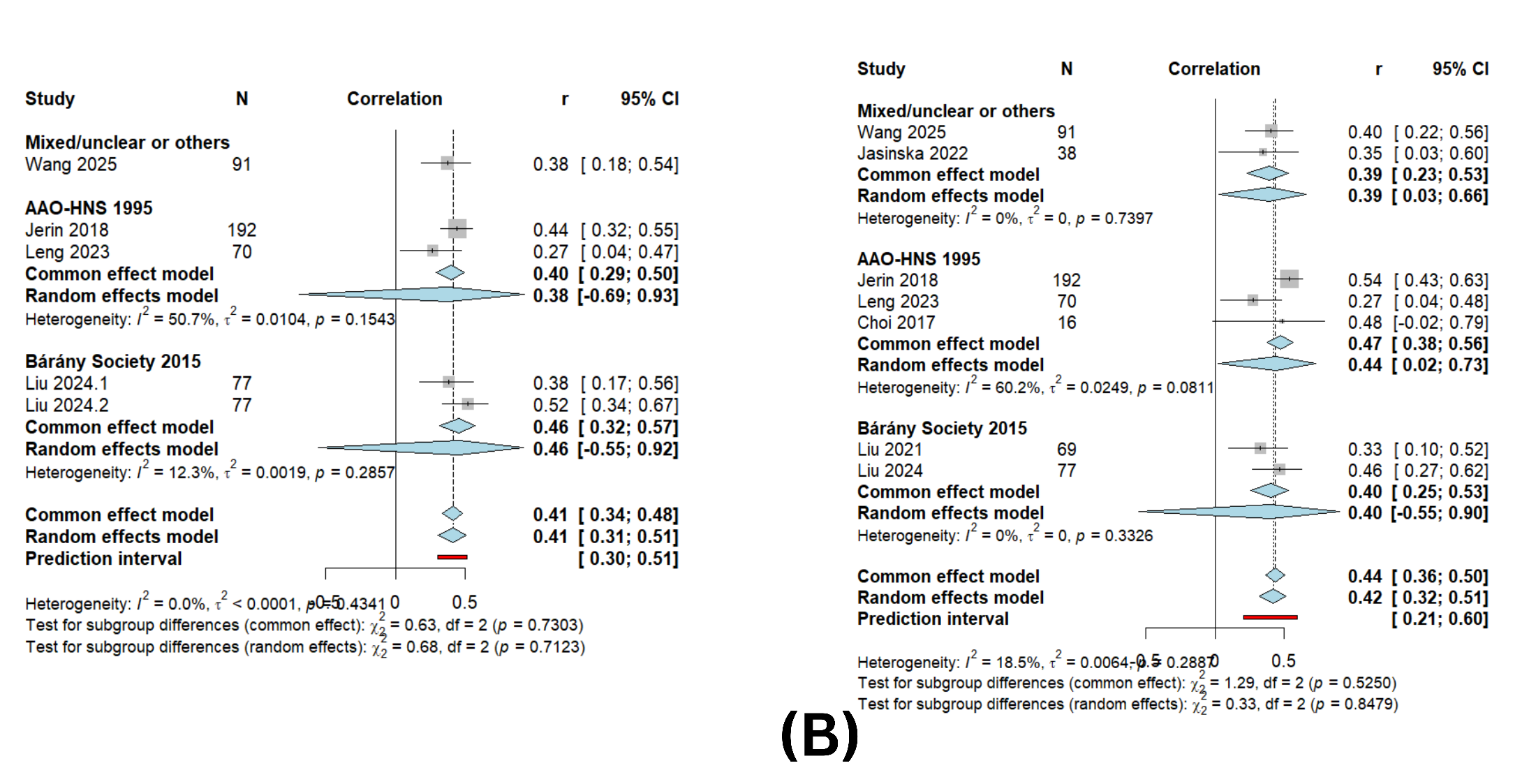


**Figure S3.** Subgroup forest plots of the correlation between EH severity and overall PTA stratified by diagnostic criteria (AAO-HNS vs Bárány): (a) cochlear EH; (b) vestibular EH.


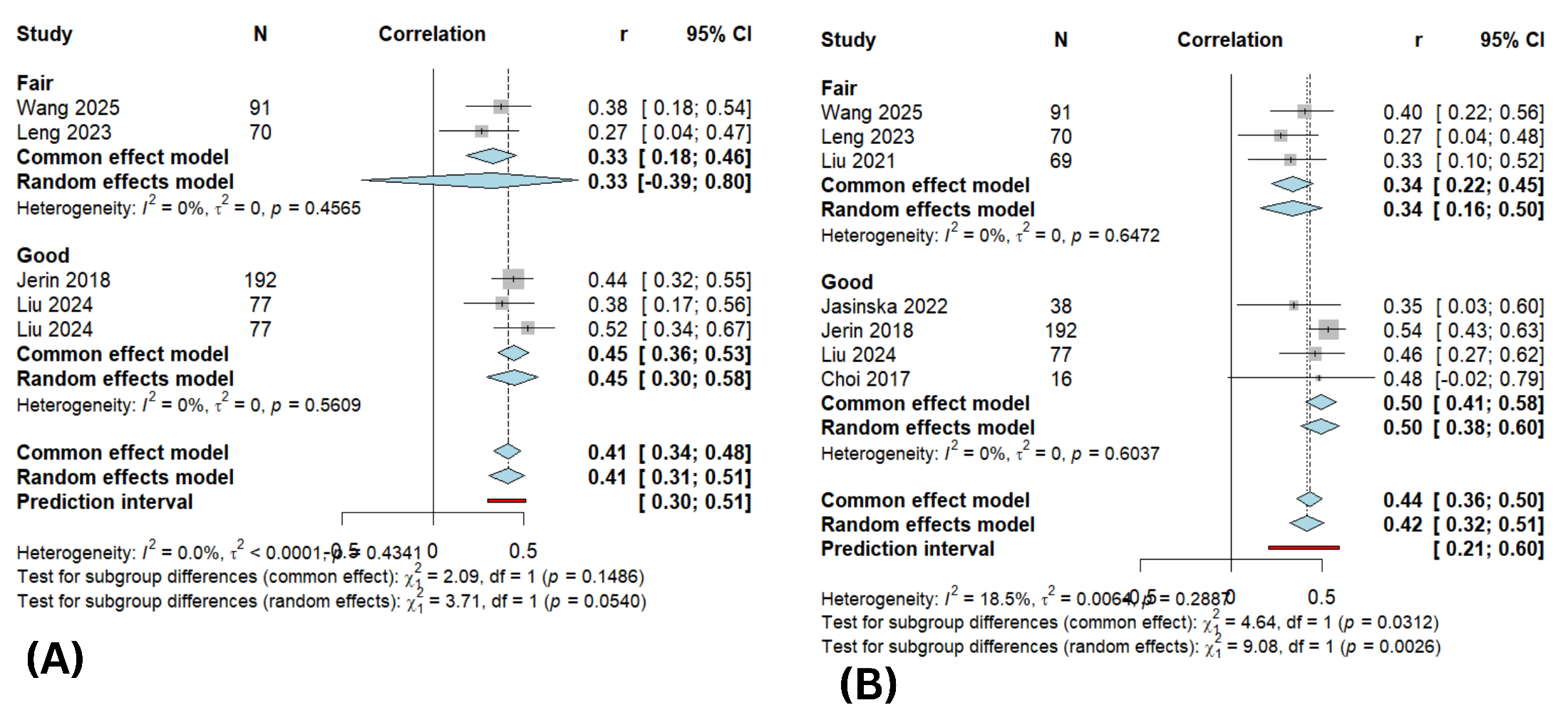
**Figure S4.** Subgroup forest plots of the correlation between EH severity and overall PTA stratified by study quality (Good vs Fair): (a) cochlear EH; (b) vestibular EH.


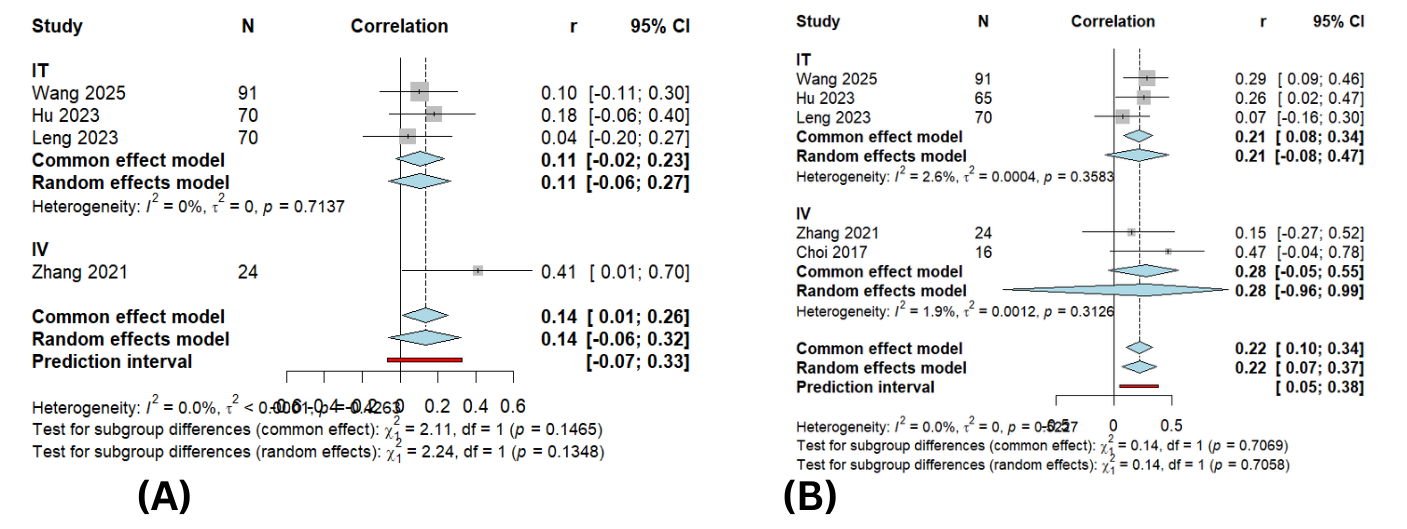


**Figure S5.** Subgroup forest plots of the correlation between EH severity and caloric canal paresis (CP%) stratified by contrast administration route (IT vs IV): (a) cochlear EH; (b) vestibular EH.


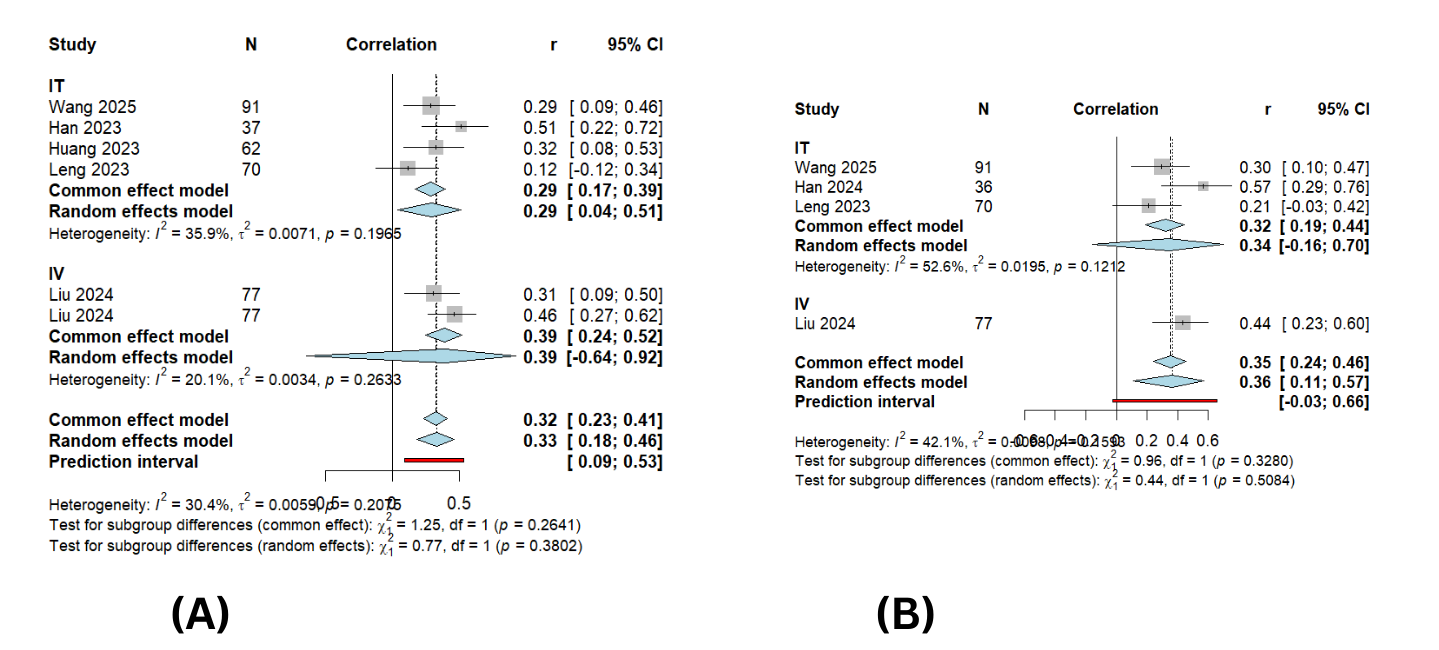


**Figure S6.** Subgroup forest plots of the correlation between EH severity and MD clinical stage stratified by contrast administration route (IT vs IV): (a) cochlear EH; (b) vestibular EH.
